# LIM Domains Target Actin Regulators Paxillin and Zyxin to Sites of Stress Fiber Strain

**DOI:** 10.1371/journal.pone.0069378

**Published:** 2013-08-21

**Authors:** Mark A. Smith, Elizabeth Blankman, Nicholas O. Deakin, Laura M. Hoffman, Christopher C. Jensen, Christopher E. Turner, Mary C. Beckerle

**Affiliations:** 1 Department of Biology, University of Utah, Salt Lake City, Utah, United States of America; 2 Department of Oncological Sciences, University of Utah, Salt Lake City, Utah, United States of America; 3 Department of Cell and Developmental Biology, The State University of New York, Upstate Medical University, Syracuse, New York, United States of America; King's College London, United Kingdom

## Abstract

Contractile actomyosin stress fibers are critical for maintaining the force balance between the interior of the cell and its environment. Consequently, the actin cytoskeleton undergoes dynamic mechanical loading. This results in spontaneous, stochastic, highly localized strain events, characterized by thinning and elongation within a discrete region of stress fiber. Previous work showed the LIM-domain adaptor protein, zyxin, is essential for repair and stabilization of these sites. Using live imaging, we show paxillin, another LIM-domain adaptor protein, is also recruited to stress fiber strain sites. Paxillin recruitment to stress fiber strain sites precedes zyxin recruitment. Zyxin and paxillin are each recruited independently of the other. In cells lacking paxillin, actin recovery is abrogated, resulting in slowed actin recovery and increased incidence of catastrophic stress fiber breaks. For both paxillin and zyxin, the LIM domains are necessary and sufficient for recruitment. This work provides further evidence of the critical role of LIM-domain proteins in responding to mechanical stress in the actin cytoskeleton.

## Introduction

While the roles of biochemical signaling have been studied extensively, mechanical force has emerged as a key regulator of protein dynamics within the cell, influencing normal development and physiology, as well as disease processes. The mechanical properties of tissues profoundly impact physiologic function. Naïve mesenchymal stem cell differentiation into neuronal, muscle or bone is in part directed by environmental stiffness [1]. The ability to sense and respond to matrix stiffness drives endothelial cell behavior in capillary morphogenesis [2] Additionally, neuronal cell differentiation and neurite branching are impacted by the stiffness of the substrate they are grown on [3,4]. Many aspects of tumor biology, including malignant behavior, are controlled by Rho-dependent actin cytoskeletal tension [5].

The actin cytoskeleton is critical for maintaining cell structure, shape and polarity as well as the mechanical properties of the cell during the highly dynamic processes of cell adhesion and migration. Cells in tissue undergo continuously changing force dynamics, and consequently must adjust their internal structure to balance internal forces to external forces. The actin cytoskeleton assembles into distinct organizational structures including branched meshwork in the leading edge of the cell and larger cables of bundled filamentous actin polymer known as stress fibers (SF). Actin SF are active mediators of force dynamics, constantly adjusting their configuration and composition to balance extracellular to intracellular forces [6,7]. SF are contractile structures containing interleaved regions rich in myosin and α-actinin. These structures generally terminate in focal adhesions (FA), integrin rich sites which mediate attachment to the extracellular matrix (ECM). The maintenance of SF requires continuous tension, and they disassemble when tension is inhibited [8].

Zyxin is a mechanosensitive protein that responds to cytoskeletons under tension [6,7,9–13]. It contains three double zinc-finger **L**in11, **I**sl-1 & **M**ec-3, or LIM domains at its C-terminus [14]. This region is essential for FA and SF targeting [15,16] and many protein–protein interactions, including associations withp130Cas [17], and CRP [14]. The N-terminus of zyxin mediates interactions with an array of actin regulatory proteins including α-actinin [16,18,19] and VASP [20,21]. Previous work demonstrated cells subjected to cyclic uniaxial stretch reinforce their actin cytoskeleton in a zyxin dependent manner. With stretch, zyxin accumulates along SF. In the absence of zyxin, SF fail to reinforce [7].

Our lab made the novel observation that actin SF undergo spontaneous stochastic cycles of thinning and repair, characterized by a transient thinning of the actin signal within a 2-5µm region accompanied by rapid lengthening of the gap [6]. Lengthening is measured by tracking fiduciary marks that flank the strain site. Most strain events extend 2-5µm in a period of less than a minute. Following this rapid lengthening and actin signal thinning, the actin signal is restored. Stress fiber strain sites (SFSS) are stabilized and repaired by a zyxin mediated complex which accumulates robustly within seconds of initiation of the strain event [6]. These sites of concentrated zyxin quickly dissipate as actin repairs and stabilizes, leaving a region of striated SF. These strain sites show no sign of substrate attachments, and accumulate no vinculin [6]. . 

In the strain site repair complex, zyxin recruits the actin crosslinker α-actinin and the actin nucleator VASP [6]. In the absence of zyxin, or when zyxin recruitment of α-actinin is blocked through use of a mutated zyxin, strain events fail to stabilize and repair, resulting in an increase in catastrophic failures of the SF [6]. Force dynamics, as measured by traction force microscopy at associated FA, show increased stress immediately prior to occurrence of the strain event. The strain event releases tension in the system as evidenced by a drop in force at associated FA [6].

Paxillin is an adaptor protein with four LIM domains that is a functionally rich and exquisitely regulated signaling nexus between integrin mediated signaling and rho-family GTPases [22,23]. As such, it serves as a relay linking adhesion state and actin cytoskeletal dynamics [24]. As with zyxin, the LIM domains of paxillin are essential for targeting to FA, specifically LIMs 2 and 3 [25]. The N-terminus of paxillin contains a variety of protein–protein interaction motifs. These include five LD domains which mediate a number of interactions, including with the structural protein vinculin, and the regulatory protein FAK [25,26]. Paxillin’s N-terminus also contains a binding site for the tyrosine kinase Src [27]. Paxillin’s binding to its diverse partners is regulated by phosphorylation of a number of tyrosine, serine and threonine phosphorylation sites [28] by a variety of kinases [23]. Paxillin’s phosphorylation state in turn regulates its interactions with other proteins [22]. Many paxillin interacters are critical for the regulation of actin dynamics [23], including, via CrkII, the CrkII-DOCK180-ELMO complex which controls cell migration through Rac activation [29,30].

Given the mechanosensitivity of paxillin in recent proteomic studies [31], and its role in the regulation of actin dynamics, we investigated the role of paxillin in the repair and stabilization of SFSS. In this work, we show that paxillin is recruited to SFSS prior to zyxin, where it provides an actin repair function that is functionally redundant to, but independent of, zyxin-mediated repair. Paxillin’s role in SF repair does not appear to rely on FAK-dependent phospho-tyrosine signaling. Furthermore we show that the LIM domains of both paxillin and zyxin are necessary and sufficient for strain site recruitment. This work represents the first description of a role for paxillin in the maintenance of SF homeostasis, wherein paxillin responds rapidly to mechanical strain, and mediates actin SF repair and stabilization.

## Results

**Figure 1:**
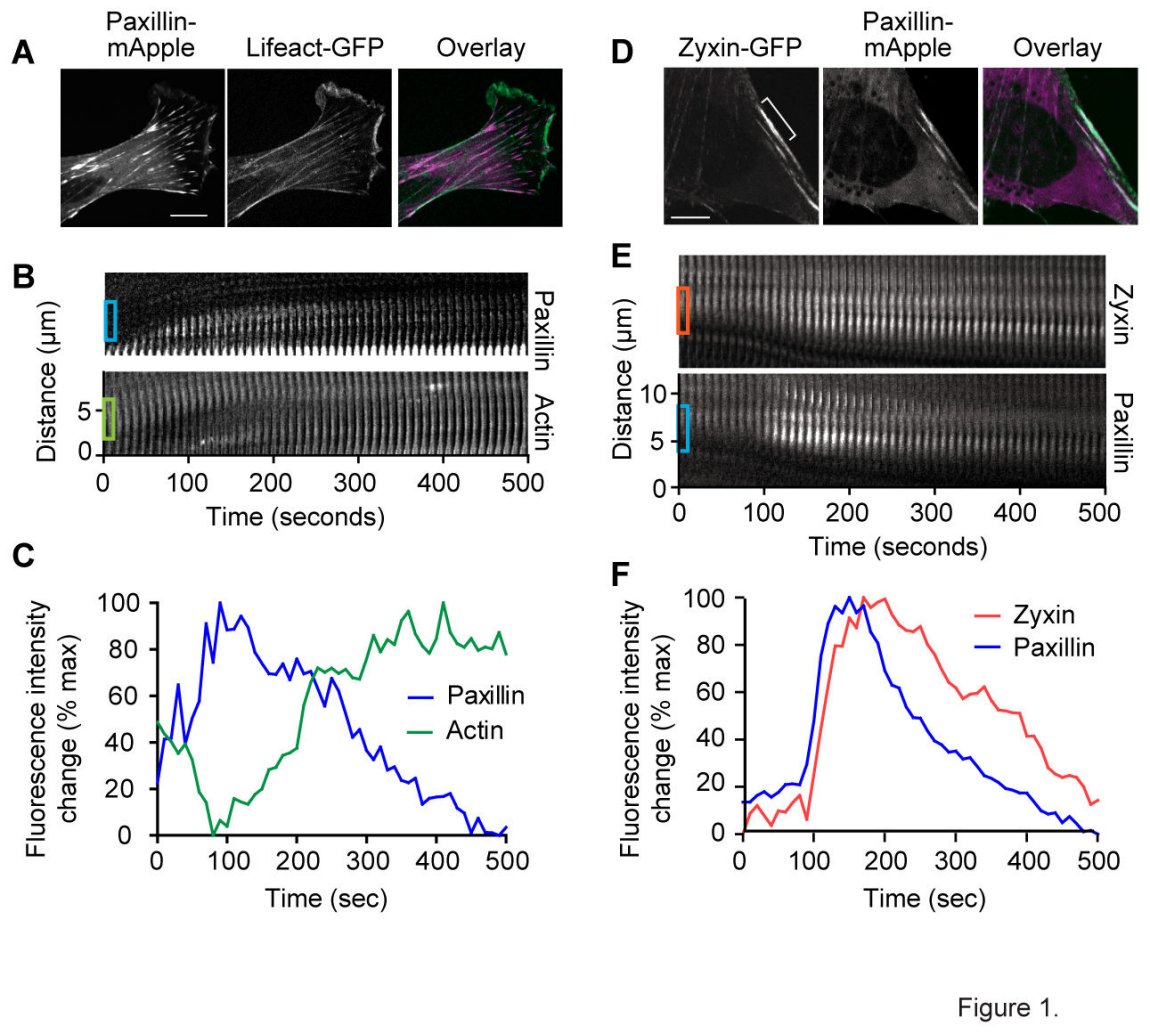
Stress fiber strain sites recruit paxillin. (A) The localization of paxillin is not restricted to focal adhesions. Time lapse micrographs taken at 10 second intervals of live mouse fibroblasts expressing paxillin-mApple and Lifeact-GFP (A and B) or zyxin-GFP and paxillin-mApple (D and E). Kymograph (B) and average intensity plotted over time (C) of boxed regions from kymograph (B) showing recruitment of paxillin and concurrent thinning actin. (D) Still frames showing overlap of paxillin and zyxin at a strain site. Kymographs (E) showing progression of zyxin and paxillin recruitment in the white bracketed region of (D) over time. Intensity plot (F) of the boxed regions on the kymograph. Scale bar=10um.

Zyxin is recruited to and is responsible for repair and stabilization of SFSS [6]. However, some repair of SFSS occurs in the absence of zyxin, indicating there must be other systems capable of strain site repair and stabilization. We hypothesized other LIM domain proteins that show mechanosensitivity at FA, such as paxillin, might also be recruited and involved in strain site repair. While paxillin is generally described as a focal adhesion component, we observed paxillin in the cytoplasm and a small proportion of paxillin to localize on SF (Figure 1A). To test whether paxillin exhibits mechanosensitive behavior on SF, we transiently transfected paxillin-mApple and Lifeact-GFP into mouse fibroblasts. Fluorescent labeled cells were plated on fibronectin coated glass coverslips and observed unperturbed using spinning disk confocal microscopy. We were able to identify spontaneously occurring strain events through thinning of actin, and localized extension of the SF. At these sites, we also observed the accumulation of paxillin with a time course and spatial restriction similar to what we observed with zyxin at strain sites. Kymographic analysis (Figure 1B and Movie S1) and intensity/time plot (Figure 1C) of the regions boxed on the kymograph (Figure 1B) show diminished actin signal coincident with paxillin accumulation.

To determine whether the sites of paxillin recruitment were also sites of zyxin recruitment, we co-expressed zyxin-GFP and paxillin-mApple in fibroblasts and followed their changing distribution using time lapse microscopy. We observed that all strain sites that recruited zyxin showed recruitment of paxillin, and all sites that recruited paxillin recruited zyxin (Figure 1D). The accumulation of paxillin occurred over roughly the same time course as zyxin, characterized by rapid accumulation and slower dissipation (Figure 1, E and F and Movie S2). However, paxillin recruitment and dissipation preceded zyxin. Additionally, paxillin accumulation occurred within the same region of the SF as zyxin (Figure 1E). Over a period of less than 10 minutes, the accumulated paxillin and zyxin had dissipated, leaving repaired, striated SF. These data suggested a role for paxillin in repair of SFSS either coordinate with, or redundant to the zyxin mediated repair system.

**Figure 2:**
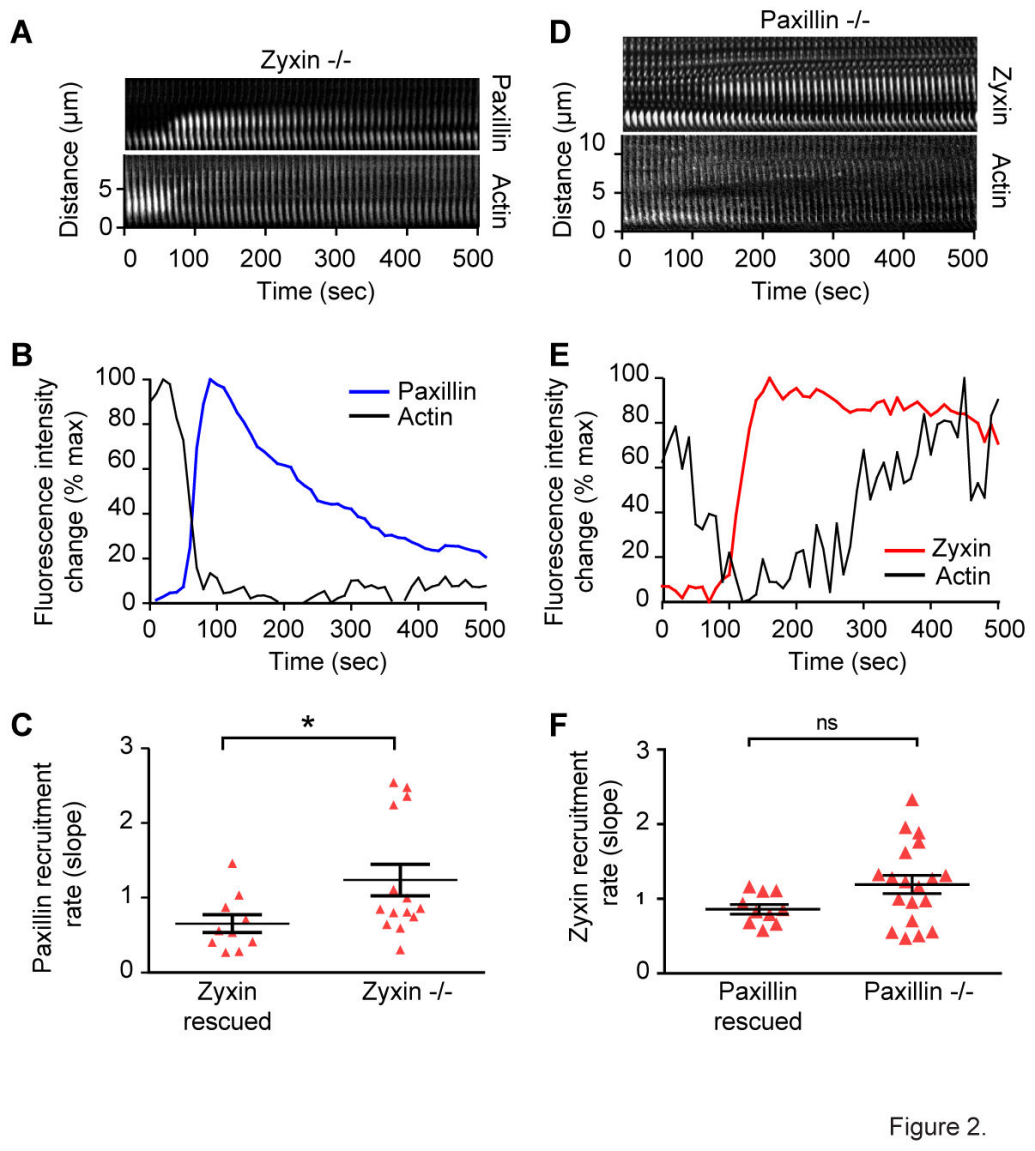
Paxillin and zyxin do not require the other for recruitment to stress fiber strain sites. Kymograph (A) and average intensity plot (B) of paxillin-GFP recruitment and actin thinning at stress fiber strain sites in zyxin-/- fibroblasts showing paxillin recruitment in the absence of zyxin. The rate of paxillin recruitment at strain sites (C) is faster in cells lacking zyxin when compared to zyxin rescued cells. Kymograph (D) and intensity plot (E) of zyxin-GFP recruitment and actin thinning at stress fiber strain sites in paxillin-/- fibroblasts showing zyxin recruitment occurs in the absence of paxillin. The rate of zyxin recruitment (F) is not significantly different in paxillin-/- cells when compared to paxillin rescued cells.

Paxillin and zyxin have not been shown to interact directly. However, their concurrent appearance at strain sites suggested that one of these proteins might be responsible for recruitment of the other. To test whether paxillin recruitment was dependent on zyxin, we transfected paxillin-GFP and Lifeact-mApple into cells isolated from zyxin null mice and watched for spontaneous strain events in unperturbed cells using spinning disk confocal microscopy. In these cells, we continued to see robust paxillin recruitment to all strain sites (Figure 2 A and B and Movie S3). In fact, the kinetics of recruitment, as indicated by fitting the slope of initial recruitment, were slightly but significantly accelerated in the cells lacking zyxin (Figure 2C), possibly due to less competition for docking sites.

To test zyxin’s dependence on paxillin for recruitment, we expressed zyxin-GFP and Lifeact-mApple in fibroblasts isolated from paxillin null mice. Again, we saw that zyxin accumulated at spontaneously occurring strain sites in the absence of paxillin (Figure 2 D and E and Movie S4). There was not a statistical difference in the kinetics of zyxin recruitment with or without paxillin (Figure 2F).

Together, these data fail to support the hypothesis that one of these proteins recruits the other. However, the similar recruitment pattern and accelerated paxillin recruitment in the absence of zyxin suggests these proteins might be sharing binding sites on the stress fiber. These data also do not rule out the possibility that paxillin and zyxin are co-recruited as part of a ternary complex.

**Figure 3:**
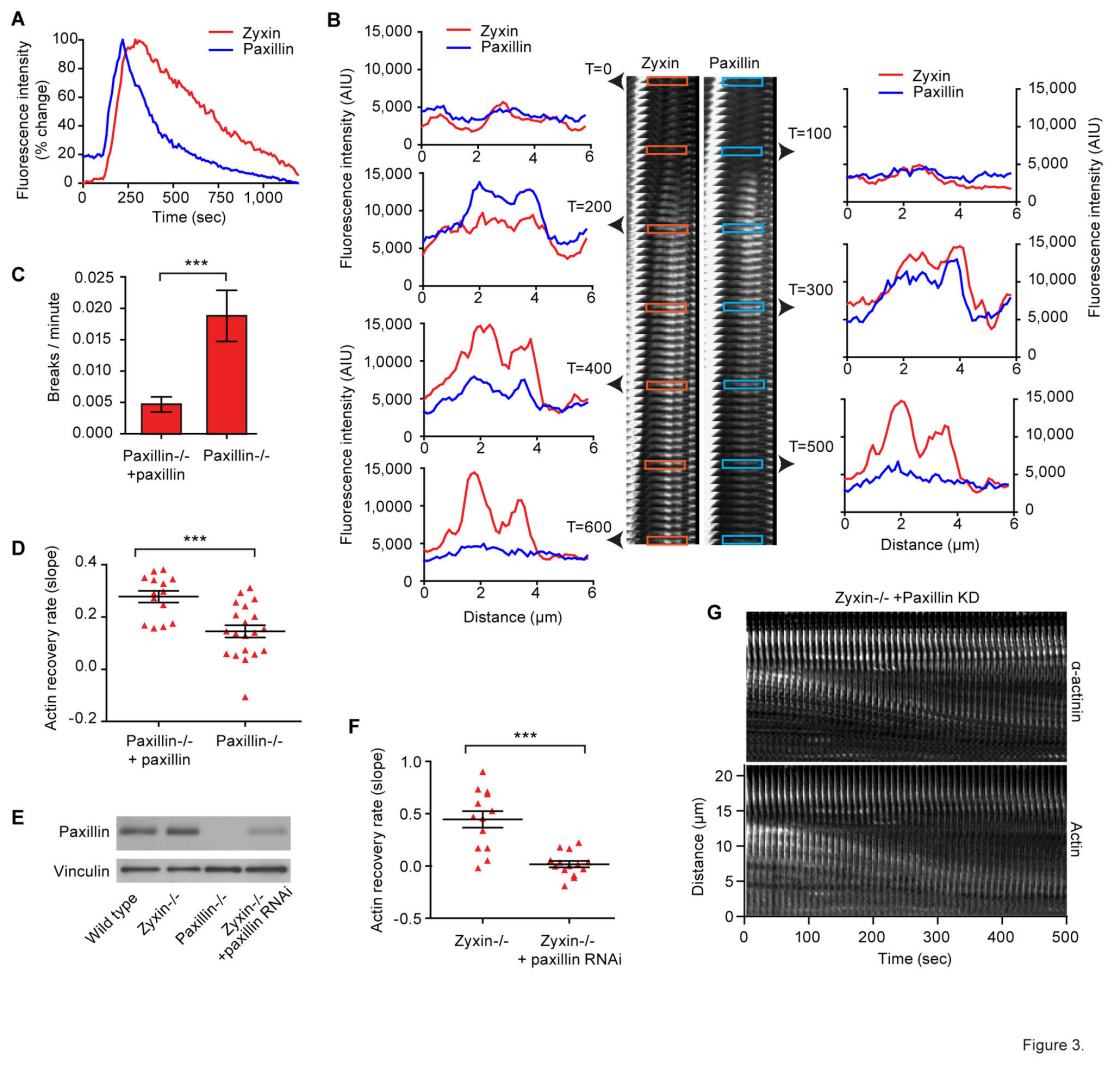
Paxillin aids stabilization of strain sites by altering actin dynamics independently of the zyxin. Comparison of normalized average paxillin and zyxin fluorescent intensity at strain site over time (A). Line scans through the strain site (B) show the spatial distribution of paxillin and zyxin fluorescence intensity (AIU) at strain sites. (C) Comparison of the catastrophic SF break frequency in paxillin null and paxillin rescued cells. (D) The actin recovery rate, as expressed by the slope of a line fit to actin intensity curve, in strain sites that do not break. (E) Western blot showing paxillin RNAi knocked down in zyxin null cells. (F) The actin recovery rate in cells lacking zyxin and with paxillin knock down. (G) Kymograph analysis of fluorescent labeled SF in zyxin null with paxillin knockdown cells. Note the SFSS slowly pull apart without repairing, but they rarely undergo catastrophic breaks within the imaging period.

If paxillin and zyxin were indeed sharing a limited set of identical binding sites within the strained region, we would expect their spatial distribution to be identical. Additionally, if the kinetics of recruitment were driven by the appearance of binding sites as a result of strain in the actin bundle, we would expect to see similar rates of recruitment for paxillin and zyxin. To study this, we looked at the relative time courses for recruitment of paxillin and zyxin in wild-type cells (Figure 3A). We found that, on average, paxillin preceded zyxin by 14 seconds. The peak of paxillin recruitment also preceded the peak of zyxin recruitment by an average of 85 seconds. To provide a high resolution analysis of the relative spatial distribution of paxillin and zyxin, we generated line scans through strain sites at 100 second intervals (Figure 3B and Movie S5). We observed that while these proteins have a similar pattern of distribution at SFSS, the points of highest intensity do not precisely overlay, and are temporally asynchronous. These data are consistent with our data indicating these proteins are recruited independently, but do not rule out the possibility that they are being recruited to the same binding sites through different mechanisms. These data also indicate that paxillin and zyxin are not part of a ternary complex that facilitates co-recruitment.

As discussed previously, zyxin helps stabilize and repair strain sites indirectly through recruitment of the actin regulators, α-actinin and VASP. However, in the complete absence of zyxin, a reduced amount of strain site stabilization and repair occurs. This indicates the presence of a second strain site repair system. To study whether paxillin might be performing this function, we compared the frequency of catastrophic SF breaks in paxillin null cells and in paxillin null cells rescued with paxillin. In the case of catastrophic breaks, a strain site initiates, but does not successfully repair, resulting in rapid and permanent retraction of the two ends. Indeed, in the paxillin null cells, we observe a four-fold increase in the frequency of catastrophic SF breaks (Figure 3C). When we analyzed the rate of actin recovery in strain sites that did not progress to catastrophic breakage, we found that actin repair was considerably diminished in the unrescued paxillin null cells (Figure 3D). These data show that, like zyxin, paxillin is involved in regulating and facilitating actin repair and stabilization within the strain site.

One possible explanation for paxillin’s role in aiding strain site repair is that it is in the same repair pathway as zyxin. If paxillin and zyxin were functioning in the same repair pathway, eliminating either would result in elimination of the repair pathway function. This predicts that elimination of either paxillin or zyxin, or elimination of both would produce the same phenotype with the same level of penetrance. If this hypothesis were correct, eliminating or reducing paxillin in cells completely lacking zyxin would have no impact on strain site repair. To test this, we used RNA interference to knock down paxillin levels [32] in zyxin null cells. We were able to achieve significant and consistent knock down (Figure 3E). We then examined strain site actin dynamics in zyxin null cells with paxillin knockdown and compared to zyxin null cells without paxillin knockdown. While most strain sites in the zyxin null cells show limited or failed repair, reduction of paxillin further degraded repair function (Figure 3F). In these cells, most strain sites were observed to slowly pull apart without ever stabilizing or breaking completely (Figure 3G and Movie S6). Since removal of paxillin further abrogates actin recovery and repair in the absence of zyxin, we conclude that paxillin is executing a complementary repair function independent of the zyxin mediated repair pathway.

Both the localization of paxillin to FA and the regulation of its protein–protein interactions at focal adhesions depend on integrin triggered signaling through focal adhesion kinase (FAK) [33]. This cascade is triggered upon cell adhesion and integrin clustering upstream of FAK dependent tyrosine phosphorylation of paxillin. Tyrosine phosphorylation of paxillin in turn is key to paxillin’s regulation, through a variety of interactions, of Rho-family GTPases and consequently actin cytoskeleton dynamics. We wanted to test whether this regulatory paradigm flowing through paxillin has a role in paxillin’s regulation of actin dynamics at SFSS. To do so, we used antibody labeling of FAK, phospho-paxillin Y118, and a non-paxillin specific antibody to detect phospho-tyrosine. To detect strain sites, we co-labeled with zyxin-GFP and Lifeact-mApple. Linescans through identified strain sites were used to assay localized antibody labeling.

**Figure 4:**
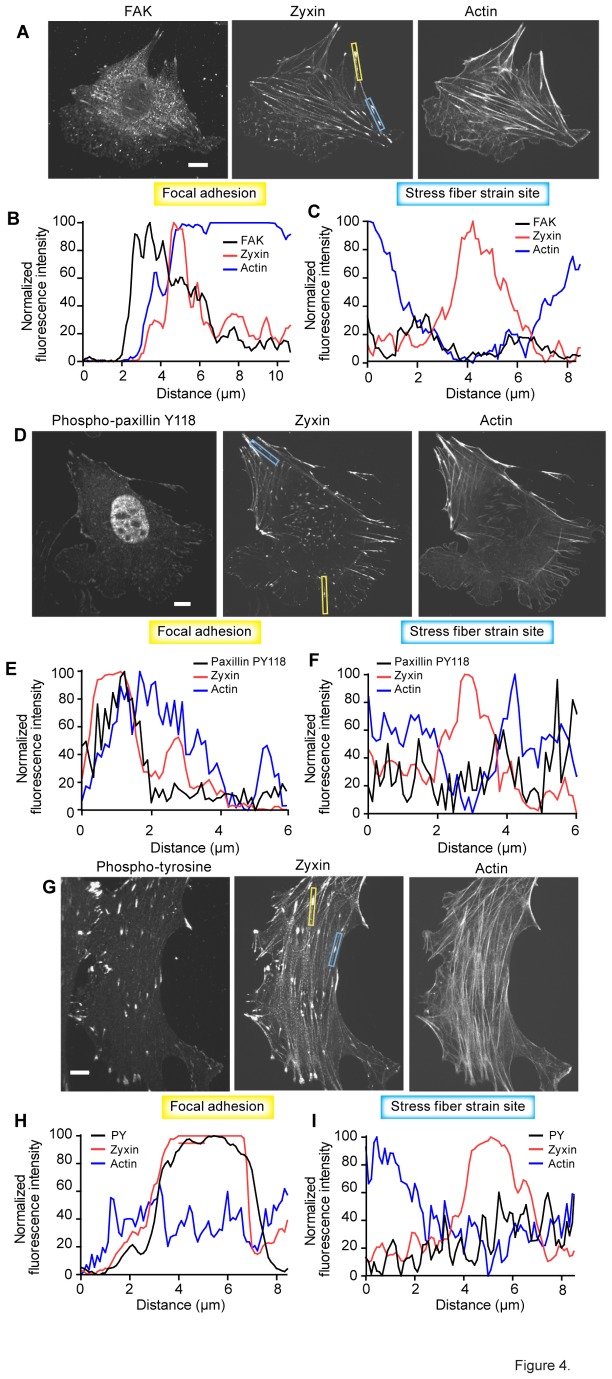
Paxillin does not stabilize stress fiber strain sites through integrin signaling. Micrographs of fixed fibroblasts labeled for paxillin interacting protein FAK in conjunction zyxin-GFP, and Lifeact-mApple (A), and linescans through either a focal adhesion (B), or stress fiber strain site (C). FAK is present in focal adhesions, but is not found at stress fiber strain sites while zyxin localizes to both sites. Micrographs of fixed fibroblasts labeled for phospo-paxillin Y118 in conjunction zyxin-GFP, and Lifeact-mApple (D). Phospho-paxillin Y118 labels focal adhesions (E) but not strain sites (F). Similarly, phospho-tyrosine immunolocalization in conjunction zyxin-GFP, and Lifeact-mApple (G) detected phopho-tyrosine activation at focal adhesions (H), but not at strain sites (I). Scale bar=10um.

If FAK was an active participant in the regulation of paxillin function at SFSS, we would expect to see significant FAK accumulation at strain sites relative to flanking regions of SF. We found strong FAK labeling at FA (Figure 4A and B), but at strain sites, as identified by limited regions of SF with high zyxin labeling and a thinned actin signal, we did not detect FAK labeling (Figure 4C). Additionally, FAK activated paxillin should be labeled by the phospho-paxillin Y118 antibody (Figure 4D). We detected PY118 at FA (Figure 4E), but not at strain sites (Figure 4F). If phospho-tyrosine activation in general were regulating paxillin function at strain sites, we would expect to see significant activation with a pan phospho-tyrosine antibody. In anti-phospho-tyrosine labeled cells (Figure 4G) we found significant labeling at FA (Figure 4H), but not at SFSS (Figure 4I). Together these results suggest that the paxillin regulation paradigm at FA is not active at strain sites.

**Figure 5:**
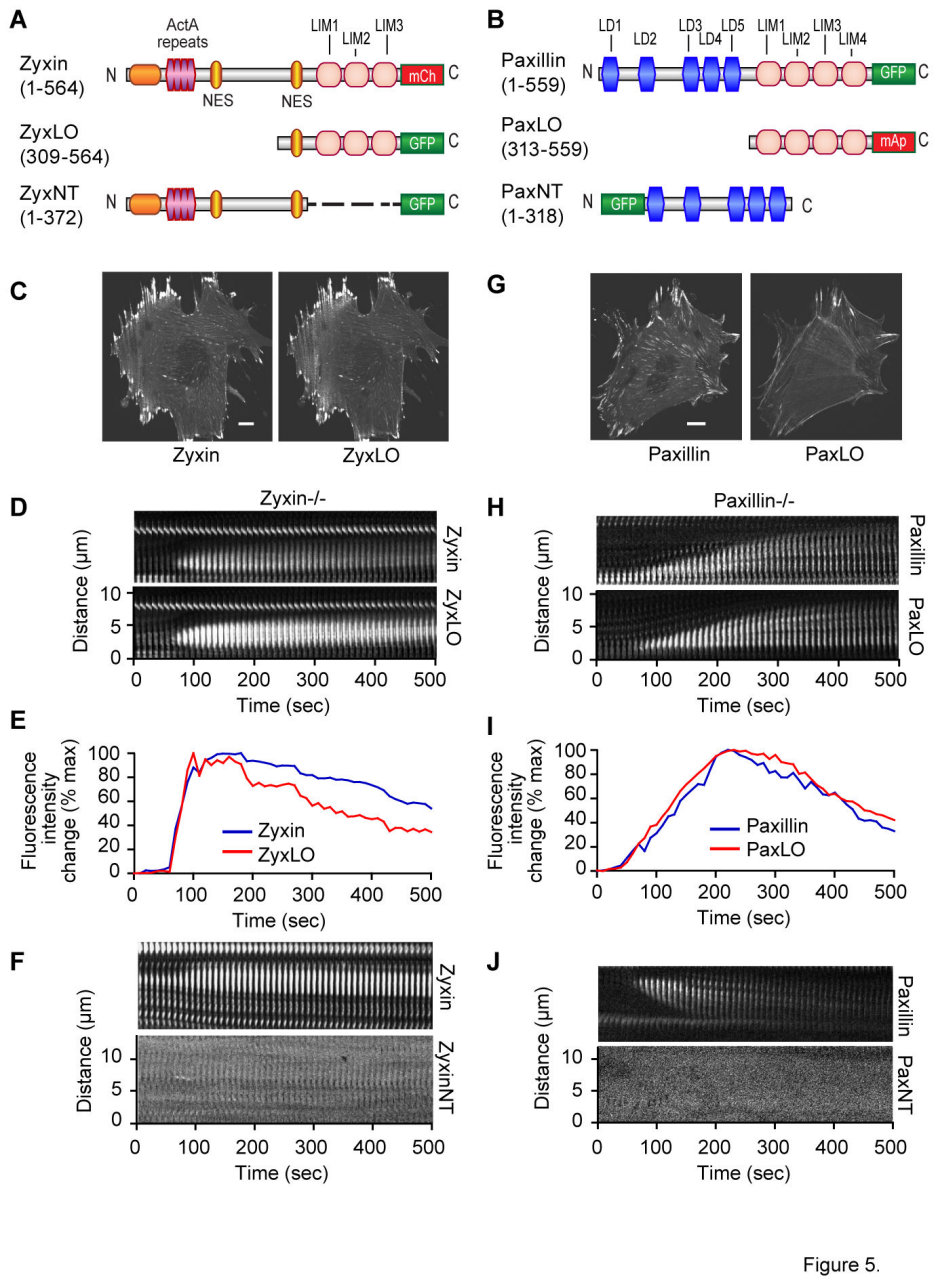
The LIM domains of paxillin and zyxin are necessary and sufficient for recruitment to SFSS. We generated GFP tagged truncated zyxin, ZyxLO (A) and mApple tagged truncated paxillin, PaxLO (B), containing the LIM domains of each protein. ZyxLO was expressed in zyxin-/- cells along with full length zyxin-mCherry (C). Time lapse imaging of ZyxLO and full-length zyxin with kymograph analysis (D) and average SFSS fluorescent intensity/time (E), show ZyxLO was recruited to SFSS in synchrony with full length zyxin. LIM-domain deleted N-terminal mutant zyxin, ZyxNT (A), did not show any strain site localization (F). PaxLO was expressed in paxillin-/- cells, along with full length paxillin-GFP. When compared to full length paxillin, PaxLO localized less to focal adhesions, and was significantly more concentrated on stress fibers (G). PaxLO was recruited to stress fibers along with paxillin (H and I). LIM-domain deleted PaxNT showed no strain site localization (J). Scale bar=10um.

As noted previously, the LIM domains of zyxin are essential for SF localization in cells subjected to uniaxial stretch [15]. Additionally, the LIM domains of paxillin are required for FA localization [25]. To test whether the LIM domains of paxillin and zyxin are also responsible for recruitment to high strain regions we generated and expressed fluorescent tagged, truncated, LIM domain only (ZyxLO and PaxLO), and LIM domain deleted (ZyxNT and PaxNT), expression systems for each protein (Figure 5A and B). ZyxLO (Figure 5A) was co-expressed with full-length zyxin-mCherry in zyxin null cells. Similarly, PaxLO-mApple (Figure 5B) was expressed along with full length paxillin-GFP in paxillin null cells. ZyxLO, as has been previously published [15], colocalized with full-length zyxin at FA and on SF (Figure 5C). ZyxLO was also observed to be sufficient for recruitment to SFSS (Figure 5D and E and Movie S7). To ensure that recruitment of ZyxLO was not being caused by a LIM-LIM interaction with full length zyxin, we repeated this experiment in cells lacking zyxin. We found that ZyxLO was still recruited to SFSS in these cells (Fig S1A). To determine whether The N-terminal region of zyxin might also have SFSS targeting function, we expressed the GFP tagged LIM-domain deleted expression construct, ZyxNT (Figure 5A) and evaluated strain site dynamics. ZyxNT showed no targeting to SFSS (Figure 5F and Movie S8). PaxLO showed diminished FA localization, but more extensive SF localization than full-length paxillin (Figure 5G). Like ZyxLO, PaxLO was also recruited to SFSS (Figure 5H and I and Movie S9). Performing this experiment in cells lacking paxillin yielded the same results (Fig S1B). Using paxillin LIM-domain deleted mutant, PaxNT (Figure 5B), we also determined that the N-terminus of paxillin lacked the capability to target SFSS (Figure 5J and Movie S10). These data demonstrate that the LIM-domains of both zyxin and paxillin are necessary and sufficient for SFSS targeting

## Discussion

Maintenance of homeostasis in the actin cytoskeleton requires sensation of mechanical stimuli resulting from migration, adhesion and tissue dynamics. In response, the cell must adjust its mechanical properties to maintain integrity within tissue and consequently the integrity of the tissue within which it resides. This complex process requires adjustment of attachment size strength and position, alteration of cortical stiffness by branched actin networks, and management of intracellular tension through the creation, remodeling and repair of linear actin networks composed of SF. SF remodeling and repair is a continuous process involving creation and elimination, thickening and thinning, as well as fracture and repair of individual fibers. This morphological evolution is required to maintain force balance in response to both intracellular and extracellular dynamics.

Our findings detailing a novel system of SF repair raise several key questions. First, how is paxillin regulating actin at SFSS? The LD domains of paxillin interact with a number of regulators of Rho family GTPases [23]. It is possible that one of these interactions is responsible for the actin repair at strain sites. For instance, the LD4 domain of paxillin interacts with the GIT-PIX-PAK-NCK complex recruiting it to the leading edge of cells [33,34], where PAK, through interaction with CDC42 and Rac1 plays a role in the regulation of cytoskeleton turnover [35]. However, this paradigm requires FAK activation of GIT2 to enable paxillin binding [33]. Considerable further work will be required to determine the mechanism by which paxillin regulates actin dynamics at strain sites.

Second, how are LIM domains targeted to sites of mechanical stress? It is important to note that while paxillin and zyxin both are recruited to SFSS, as shown here, and both exit FA when myosin II is inhibited and tension is lost [31], their response to cyclic uniaxial stretch is quite different. With stretch, zyxin, and specifically zyxin LIM domains [15], exits the FA and concentrates along SF [7], but paxillin remains at FA [36]. Hic-5 is the closest homolog to paxillin [37]. Hic-5 and paxillin participate in many of the same binding interactions including with vinculin and FAK [38], and PTP-PEST [39]. In spite of these similarities, Hic-5 is robustly recruited through its LIM domains to SF in response to stretch while paxillin is not [36]. This indicates there is a high degree of specificity to LIM domain mechanoresponse.

It also remains unclear what alteration of the SF marks it for repair. Neither paxillin nor zyxin has been shown to interact directly with actin. While it is possible that a conformational change in strained actin exposes cryptic binding sites, there is no evidence for this. SFSS are rich in free actin barbed ends [6] from broken filaments. These may provide the docking site for an actin binding protein that recruits paxillin and/or zyxin. Determining the targeting sequence within the LIM domains for each of these proteins will inform the identity of the molecular mark at the site of stress.

LIM-domain proteins are emerging as a distinct class of mechanosensors. Recent proteomic studies have suggested that a significant subset of LIM-domain proteins is concentrated at FA, and 21 of these, including zyxin and paxillin, are mechanosensitive as evidenced by their differential FA retention when myosin II dependent contractility is modulated by blebbistatin treatment [31]. Additionally, previous studies have shown that LIM-domain proteins zyxin [7], Hic-5 and CRP2 [36] are driven onto SF by uniaxial stretch. While the LIM domain double zinc-fingers provide a stable platform for interaction, the specificity of binding is conferred by shorter stretches of sequence within the fingers [40]. For both paxillin and zyxin the sequence within the zinc fingers that is essential for targeting, and the proteins that dock them remain unknown. Given the extraordinarily complex array of proteins and protein–protein interactions at FA [41], it may prove useful to the understanding of LIM-domain protein mechanoresponsiveness to study that response within the simpler set of proteins at SFSS.

Our results show that paxillin, through its LIM-domains, is recruited to high strain sites on SF. At these sites, paxillin is involved in repair that facilitates the recovery of actin and consequent stabilization of the strain site. This repair is carried out in the absence of FAK activation of paxillin. This process of recruitment and repair is parallel to, but independent of, the zyxin repair system. The work presented here, showing the rapid recruitment of two independent LIM-targeted repair systems provides additional evidence that the role of LIM domains in mechanosensation is not restricted to FA.

## Materials and Methods

### Cell lines

Production and immortalization of fibroblasts derived from wild-type and zyxin-null mice was described previously [42]. Fibroblasts derived from zyxin (-/-) mice were stably rescued with N-terminally tagged zyxin or mutant zyxin by viral infection followed by FACS sorting to select cells expressing fluorescently tagged zyxin [15].

### Cell culture and transfection

Cells were cultured in DMEM supplemented with L-glutamine, penicillin/streptomycin, sodium pyruvate and 10% fetal bovine serum (Hyclone) and grown on coverslips coated with fibronectin (10 µg/ml.) Transient transfections of DNA constructs for expression of fluorescently tagged proteins were performed using FuGene HD transfection reagent (Promega) or Lipofectamine 2000 transfection reagent (Invitrogen). Time lapse imaging of cells was performed 2-6 days after transfection.

### DNA constructs

#### pZyx-mCherry

The mCherry coding sequence [43] was PCR amplified to add BamHI and NotI restriction sites. The PCR products were digested in BamHI and NotI and then ligated into pEGFP-zyxin, replacing the EGFP coding sequence.

#### pLenti-Paxillin EGFP and pcDNA-Paxillin mApple

Cloned using Invitrogen’s Multisite Gateway Cloning. An L1R5 Paxillin entry clone was created by PCR amplification of chicken Paxillin with a 5’ attB1 and 3’ attB5r recombination site. PCR product was recombined with pDONR221 P1P5r plasmid (Invitrogen) to create the L1R5 Paxillin entry clone. An L5L2 Gateway entry clone was made for both mApple and EGFP by PCR amplification with a 5’ attB5 and 3’ attB2 recombination site. PCR product was recombined with pDONR221 P5P2 plasmid (Invitrogen) to create the entry clone. The Paxillin entry clone was recombined with the mApple entry clone and pcDNA6.2-DEST plasmid (Invitrogen) to create the pcDNA-Paxillin-mApple expression clone. The Paxillin entry clone was recombined with the EGFP entry clone and pLenti6.3-DEST plasmid (Invitrogen) to create the pLenti-Paxillin-EGFP expression clone.

#### pLenti-Lifeact-mApple and pLenti-Lifeact-EGFP

Cloned using Invitrogen’s Multisite Gateway cloning. EGFP/mApple were amplified by PCR with a 5’ attB1 and 3’ attB2 recombination site. To the 5’ attB1 primer the 51bp Lifeact sequence (atgggtgtcgcagatttgatcaagaaattcgaaagcatctcaaaggaagaa) was added to incorporate Lifeact directly upstream of either the mApple or EGFP sequence. PCR product was recombined with a pDONR221 plasmid (Invitrogen) to create the L1L2 entry vector. This was then recombined with pLenti6.3-DEST to create the expression plasmid.

### RNA interference

Custom Silencer siRNA oligos (Invitrogen/Ambion) targeting the sequence CAAGCAGAAGUCGGCAGAG of paxillin [32] were transfected into Zyxin -/- cells using Oligofectamine (Invitrogen). 24 hours post RNAi transfection cells were plated on glass and transiently transfected with pcDNA α-actinin GFP and apple actin. Cells were then imaged 48 hours post RNAi transfection.

### Live-cell imaging for protein dynamic studies

Coverslips were mounted in an open magnetic imaging chamber (Chamlide), with DMEM/F12 media (Invitrogen) supplemented with L-glutamine, penicillin/streptomycin, sodium pyruvate and 10% fetal bovine serum. Cells were maintained at 37^0^C using a stage-top microscope incubator (Pathology Devices). Imaging was performed on an Andor spinning disk confocal on an inverted Nikon Ti-E microscope with a 60X 1.49NA Nikon, Plan Apochromat TIRF lens. Illumination was from solid state 488nm and 568nm lasers (Melles Griot), switched by an acousto-optic tunable filter based laser combiner (Andor Technology), and delivered by optical fiber to the Yokogawa CSU-10 scanhead. The emission light path was equipped with switched bandpass filters (Semrock Inc). All time-lapse image sequences were captured at 10 second intervals using Andor DV887 1024X1024 EMCCD camera (Andor Technology). Stage motions were controlled in XY with a motorized XY stage (Prior Instruments) and in Z with an integrated Perfect Focus system (Nikon). Image acquisition was performed using Andor IQ imaging software (Andor Technologies) on a PC workstation (HP Computers).

### Immunofluorescence microscopy

Zyxin -/- cells stably expressing GFP Zyxin were transiently transfected (Promega FuGENE HD) with apple actin and plated on ΔT dishes (Bioptechs). 48 hours post transfection cells were fixed (15 min, 3.7% formaldehyde) and permeabilized (3min, 0.5% triton X-100), blocked (1hr, 10% NGS/3% BSA), then probed with either FAK (c-20) antibody at 1:50 (Santa Cruz) or PY 4G10 at 1:50 (Millipore) or paxillin PY118 at 1:100 (Invitrogen). Localization was detected with Alexa Fluor 405 secondary antibody (Molecular Probes).

### Image processing and analysis

#### Fluorescence intensity measurements

Image sequences were processed, region intensity and distance measurements were collected, and movies were generated using MetaMorph software (Molecular Devices). Intensity measurements were taken as average intensity within a region of interest restricted to the site of SF elongation. Kymographs were generated using a custom macro (Ryan Littlefield) run in MetaMorph. For these, a 10 pixel wide linear region of interest along a SF was selected, the image was rotated using the nearest neighbors rotation algorithm to eliminate diagonal pixel sampling, and then each 10 pixel region was output into a montage. Numerical output was normalized and graphed using Prism 5 (GraphPad).

#### Analysis of actin recovery

We tracked actin-mApple or Lifeact-GFP or Lifeact-mApple fluorescent intensity within the strain region. Starting from the low point in the intensity plot, as the region entered the repair phase, we fit a line to the trajectory of recovery, restricted to the first 200 seconds of recovery, then calculated recovery rate (fluorescence intensity/time) based on the slope of this line.

#### Statistical analysis

All statistical analysis was performed using Prism 5 (GraphPad). Statistical significance for the analyses of SF breaks, changes in traction induced strain, the kinetics of fluorescence accumulation, and the kinetics of fluorescence dissipation were determined using unpaired, two-tailed t-tests. Contingency analyses of actin recovery, and paxillin and zyxin recruitment utilized a two-tailed Fisher’s exact test. Differences were considered significant at the 95% confidence level. Statistical significance denoted as follows; *** p<0.001, ** p=0.001 to 0.01, * p=0.01 to 0.05.

### Western Blots

10µg cell lysates were harvested using SDS sample buffer and run on SDS-PAGE with Precision Plus molecular weight markers (Bio-Rad). Blots were probed with Paxillin antibody at 1:5,000 (BD 349 paxillin) and vinculin antibody 1:20,000 (Sigma hVIN-1). Detected using HRP-conjugated secondary antibodies (GE Healthcare) and ECL (GE Healthcare).

## Supporting Information

Figure S1 **ZyxLO-GFP was co-expressed with Actin mApple in cells completely lacking full length zyxin.** Kymograph analysis of SFSS showed robust accumulation of ZyxLO (A). Additionally, PaxLO mApple was co-expressed with Actin-GFP in cells completely lacking paxillin. Kymograph analysis of SFSS continued to show accumulation of PaxLO.
(TIF)

Movie S1 **Associated with Figure 1B.** Shows concurrent paxillin recruitment to sites of actin thinning.
(AVI)

Movie S2 **Associated with Figure 1E.** Shows that zyxin and paxillin are both recruited to SFSS.
(AVI)

Movie S3 **Associated with Figure 2A.** Shows paxillin is still recruited to SFSS in the absence of zyxin.
(AVI)

Movie S4 **Associated with Figure 2D.** Shows zyxin is still recruited to SFSS in the absence of paxillin.
(AVI)

Movie S5 **Associated with Figure 3B.** Shows that while paxillin and zyxin are concurrently recruited to SFSS, their time courses of recruitment are not identical.
(AVI)

Movie S6 **Associated with Figure 3G.** Shows lack of SFSS stabilization in zyxin null, paxillin knock down cells.
(AVI)

Movie S7 **Associated with Figure 5D.** Shows concurrent recruitment of full-length zyxin and LIM-domain only zyxin (ZyxLO) to SFSS.
(AVI)

Movie S8 **Associated with Figure 5F.** Shows LIM-domain deleted zyxin (ZyxNT) is not recruited to SFSS with full-length zyxin.
(AVI)

Movie S9 **Associated with Figure 5H.** Shows concurrent recruitment of full-length paxillin and LIM-domain only paxillin (PaxLO) to SFSS.
(AVI)

Movie S10 **Associated with Figure 5J.** Shows LIM-domain deleted paxillin (PaxNT) is not recruited to SFSS with full-length paxillin.
(AVI)
