# Supplementary figures and images for: LIM Domains Target Actin Regulators Paxillin and Zyxin to Sites of Stress Fiber Strain
Source: PLoS One. 2013 Aug 21;8(8):e69378. doi: 10.1371/journal.pone.0069378 (PMC3749209; doi:10.1371/journal.pone.0069378)

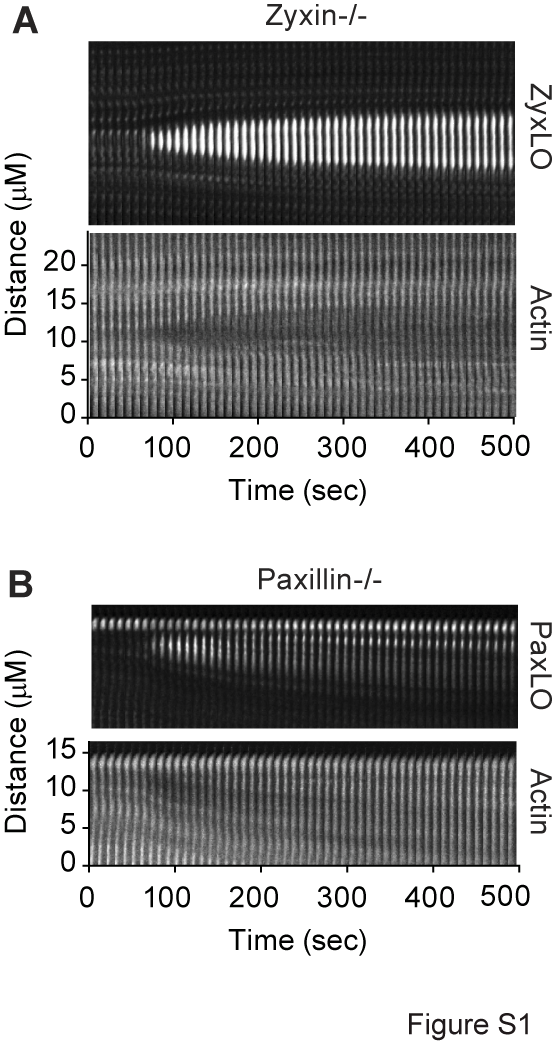

Supplement: Figure S1 — ZyxLO-GFP was co-expressed with Actin mApple in cells completely lacking full length zyxin. Kymograph analysis of SFSS showed robust accumulation of ZyxLO (A). Additionally, PaxLO mApple was co-expressed with Actin-GFP in cells completely lacking paxillin. Kymograph analysis of SFSS continued to show accumulation of PaxLO. (TIF) [file pone.0069378.s001.tif]
